# Collaborative relationships in translational medical research among Chinese clinicians: an internet-based cross-sectional survey

**DOI:** 10.1186/s12967-021-02911-5

**Published:** 2021-06-05

**Authors:** Meina Li, Bin Lian, Xiaoxiong Xu, Pan Zhao, Bihan Tang, Chaoqun Hu, Xiang Liu, Wenya Yu, Lulu Zhang

**Affiliations:** 1 Department of Military Health Management, College of Health Service, Second Military Medical University, 800 Xiangyin Rd, Shanghai, 200433 China; 2 School of Public Health, Shanghai Jiao Tong University School of Medicine, 227 South Chongqing Rd, Shanghai, 200025 China; 3 The Affiliated Suzhou Science &, Technology Town Hospital of Nanjing Medical University, Suzhou, 215153 China; 4 Navy Medical Center, Shanghai, 200000 China; 5 The Fifth Medical Center, Chinese PLA General Hospital, Beijing, 100039 China; 6 Department of Respiratory Disease, The 903Rd Hospital of PLA, Hangzhou, 310000 China

**Keywords:** Translational medicine, Translational medical research, Translational research, Collaboration, Clinician, China

## Abstract

**Background:**

This study aimed to explore the collaborative relationship in translational medical research from the perspective of clinicians in China. The findings are expected to help practitioners optimize and experience the greatest advantages of collaboration.

**Methods:**

We conducted a national internet-based survey from July 29 to October 12, 2020. Of the 806 responses, 804 were completed with valid responses (valid response rate = 99.8%). The collected data were presented as descriptive statistics and analyzed using nonparametric tests (including the Wilcoxon rank test and Kruskal–Wallis H test) and stepwise logistic regression.

**Results:**

Of the 804 participants, 733 were either willing or very willing to collaborate in translational medical research. Clinicians’ willingness was influenced by their current research type, role in current translational medical research, burdens of their present research, preferred partners for collaboration at the institutional or individual level, and preferences for independent or dependent relationships.

**Conclusions:**

Clinicians should evaluate their time, role, burdens, personal preferences for research relationships, and appropriate partners based on their current translational medical research and its goals, before deciding to collaborate.

## Background

Translational medical research (TMR), which aims to bridge laboratory and clinical research [1], has experienced rapid development in the past 20 years [2]. In TMR, collaboration has become mainstream and is now a fundamental requirement. TMR consists of a continuous process that begins with clinical discovery and medical research and leads to clinical application in patients [3]; it accommodates multiple disciplines and scientists in various fields. However, owing to the professional gap between laboratory scientists and clinicians and the differences among hospitals, universities, research centers, and industries [4–6], practitioners need to collaborate at an individual or institutional level to realize the goals of TMR.

Researchers have considered in depth the advantages and disadvantages of collaboration in TMR. Collaboration, especially in a multidisciplinary team, is widely recognized to be of great significance and necessity in TMR [7]. Although “translation” has different meanings for different stakeholders, the overall aim is to benefit humankind by promoting the application of clinical findings and laboratory achievements. Therefore, collaboration among clinicians, laboratory scientists, industrial scientists, biotechnologists, and politicians in different institutions can bring these stakeholders together, help them understand one another professionally, and motivate TMR [8, 9]. Among all the merits of collaboration in TMR, the most prominent is knowledge exchange, which can help elucidate scientific questions, promote laboratory research, and accelerate clinical application [3, 10–14]. A bibliometric research further confirmed the role of collaboration in TMR, especially in terms of knowledge exchange [15]. Therefore, based on such knowledge exchange and mutual understanding, collaboration between researchers and clinicians is crucial for the translation and application of laboratory biomedical discoveries to patients and to clinical practice [16–19]. Additionally, collaboration between researchers and industrial scientists can promote the development of medicine [20].

Collaboration in TMR also has its disadvantages and risks. For instance, collaboration in TMR among various partners could lead to greater time expenditure or delay of research plans, owing to diverse academic perspectives or scholarly disagreements [3, 21]. Collaboration also tends to cost more, including the time spent in negotiation and exchange, as well as the costs of communication and travel, contradictions in research design and results, and conflicts arising from result sharing [3, 14]. Some stakeholders also refuse to collaborate in TMR because of the risks of losing decision-making autonomy and frustration with partners [3].

Considering the characteristics of collaboration in TMR, different practices have been observed, including collaborations at the institutional and at individual levels [7]. In China, current collaborations in TMR mainly focus on the institutional level, such as the Sino–Cuban Joint Working Group on Biotechnology Cooperation [22], and the collaboration between Peking University Health Science Center and the University of Michigan Medical School [23]. However, from the perspective of individuals, their attitudes, viewpoints, willingness, and practice behaviors are important for collaboration in TMR; this is especially true for clinicians, who are situated at the juncture of laboratory and clinical research. This study therefore explored collaborative relationships in TMR from the perspective of clinicians to determine their willingness to collaborate, recognize preferred partners, and determine the factors influencing collaboration. The results of this study provide key evidence for optimizing collaboration and realizing the greatest advantages of collaboration in TMR.

## Methods

### Study design

We designed an internet-based survey to explore the collaborative relationships in TMR among clinicians in China. The required sample size was determined to be at least 129, which was calculated with a confidence level of 95%, an admissible error of 0.1, and the probability of approval of TMR among clinicians of 74.9% [2]. A preliminary investigation involving 85 clinicians was conducted before the formal survey. Based on the preliminary participants’ responses, several items were revised to improve the reliability and validity of this questionnaire. The internal consistency of the formal questionnaire was examined using a Cronbach’s α coefficient, which was calculated as 0.930, indicating good reliability. Factorability was tested using the Kaiser–Meyer–Olkin test and Bartlett’s test of sphericity, which yielded values of 0.724 and 5103.91 (*p* < 0.0001), respectively, suggesting good validity.

The formal questionnaire included 24 items that were compiled through a review of references and consultations with experts. Information on demographic characteristics, current status of personal TMR, collaborative willingness in TMR, and perceptions of collaboration in TMR was collected. Items that collected participants’ perceptions were rated using a five-point Likert scale.

Regarding demographic characteristics, we analyzed the clinicians’ sex, age, educational level, professional title, and department. Regarding the current status of personal TMR, we asked about the clinicians’ research type (e.g., clinical, laboratory, or public health management), role in TMR (e.g., principal investigator [PI] or participant), research pressure (low or high), and communication methods used in collaborations (e.g., face-to-face, telephone, or WeChat). The scale of collaborative willingness in TMR contained five items, including willingness to collaborate and preferred collaboration partners at an institutional or individual level. Perceptions of collaboration in TMR were addressed via 11 items that focused on collaborative relationships, positive and negative aspects of collaboration, and factors that influence collaboration. The measure of collaborative relationships explored the preferences for independent or interdependent relationships [24].

Referring to the positive aspects of collaboration, items addressed understanding of collaboration advantages, extra resources made available through collaboration, and improved personal capabilities. The advantages of collaboration included additional funds or resources, knowledge transfer, enhancement of reputation, increase in number of publications, improvement in publication quality, enrichment of academic influence, additional clinical resources, more equipment resources, new technologies, promotion of treatment capability, and acceleration of the research process [3, 9, 25]. Extra resources made available through collaboration referred to funds, patients, technologies, equipment, talents, and information. Personal capabilities could be improved in terms of communication, receiving new knowledge and technology, and control over research programs.

When considering the negative aspects, we focused on the disadvantages such as the costs, risks, and challenges of collaboration. Disadvantages included more time spent on communication, personal resource transfer to partners, loss of research autonomy and control, deviation from one’s main research, and conflicts regarding key research points [3, 26]. Collaboration costs included the costs of selecting partners and collecting information, negotiation, implementation, and supervision [9]. The risks of collaboration were identified as the risks associated with coordinating all partners, an imbalance of duties and responsibilities among partners, and dropping out or breaking of promises by partners [27]. The challenges faced during collaboration referred to competition from other research organizations, the ethics review process, insufficient research funds, and the recruitment of project managers [28].

Factors that influence collaboration included those related to the implementation and the success of the collaboration. The former involved factors such as geographical locations, funds, technologies, information, academic status, mutual relationships, and partners’ cooperation patterns [29]. The latter included explicit collaboration aims, specific collaboration periods, appropriate partners, clear collaboration rules, clear-cut benefit distribution rules, explicit risk-taking rules, maintaining cooperative network relationships, establishing coordination and supervision mechanisms, specific penalty rules for violations of the collaboration agreement, and definite rules for dealing with disputes or emergencies [30, 31].

### Data collection

The formal internet-based investigation was conducted from July 29 to October 12, 2020. Of the 806 questionnaires distributed at random to clinicians nationally, 804 were returned with valid responses (valid response rate = 99.8%). The inclusion criteria were (1) clinicians should have TMR experience, (2) all clinicians should voluntarily participate, (3) participating clinicians should complete the survey online and provide informed consent. Those who were not clinicians, did not have TMR experience, or could not complete the online survey were excluded from this investigation. Only completed questionnaires could be submitted to the online survey system.

### Statistical analyses

Our statistical analyses were performed using SAS 8.2 (SAS Institute Inc., Cary, NC, USA) and PASW Statistics for Windows, Version 18.0 (SPSS Inc., Chicago, IL, USA). The descriptive statistics, univariate analysis, and multivariate analysis were implemented step by step. First, we employed frequency and percentage to obtain detailed descriptions in the descriptive statistics. Second, the univariate analysis was conducted to confirm the influence of one factor on collaborative willingness in TMR. If *p* < 0.05, the influence was statistically significant. Third, by including all factors with statistical significance in the univariate analysis, we used a stepwise logistic regression model to determine the influence of multiple factors on collaborative willingness in TMR. In particular, descriptive statistics were used to describe the basic characteristics of the participants. Nonparametric tests, including the Wilcoxon rank test and Kruskal–Wallis H test, were used to test participants’ willingness to collaborate in TMR. A stepwise logistic regression analysis was used to analyze the factors influencing the willingness to collaborate in TMR, with inclusion and exclusion criteria of 0.10 and 0.15, respectively. All tests were two-tailed, with *p* < 0.05 considered to be statistically significant.

Based on the results of the univariate analyses, only factors with a statistically significant influence on collaborative willingness in TMR among clinicians were included in the logistic regression analysis.

## Results

### Basic characteristics of participating clinicians

Of the 804 participants, 57.5% were men and nearly half were 31–40 years old. Most had a master’s degree and an intermediate professional title and worked in non-surgical departments. More than a third (38.1%) of participants were engaged in clinical studies at the time of the survey and most acted as a project participant (not PI). Almost half of participants reported a relatively heavy research burden (based on the five-point Likert scale). The most popular communication method was WeChat (an instant messaging application), with 63.8% reporting using it “often” or “always.”

More participants preferred collaborating with individuals rather than with institutions, and the most popular institution type was research institutes. However, their preferences for individuals and institutions varied across different stages of the research. In the research application stage, the most preferred institutions and individuals were research institutes and laboratory scientists, respectively. In the research implementation stage, preferences were for collaboration with research institutes and clinical scientists. In the research achievement promotion stage, preferences were for industrial partners and industrial staff. The number of clinicians who favored independent relationships was nearly equal to those who favor interdependent relationships.

Among the advantages of collaboration, improvement in reputation was the most recognized (93.8% agreed or strongly agreed), followed by improved publication quality (93.5%), promotion of knowledge transfer (92.8%), and increased number of publications (92.8%). Information resources were the most recognized extra resources made available by collaboration. The personal capability to receive new knowledge and technology was considered the most positive influence of collaboration.

Of all the disadvantages of collaboration, the transfer of personal resources was considered to be the worst, with 60.1% of the participants agreeing or strongly agreeing. Most participants recognized that the cost, risk, and challenge of collaboration were the costs of selecting partners and collecting information (89.8%), the risks of unbalanced duties and responsibilities among partners (87.5%), and the challenge of recruiting project managers (87.4%), respectively.

The professional technological input of partners was the factor that most influenced desire to collaborate; 92.8% of participants expressed agreement in this respect. For successful collaboration, having explicit collaboration aims (93.4%) and appropriate partners (93.4%) were considered equally important (Tables 1 and 2).

**Table 1:** Basic characteristics of participating clinicians

Characteristic	N (%)
Sex	
Male	462 (57.5)
Female	342 (42.5)
Age (year)	
20–30	220 (27.4)
31–40	399 (49.6)
41–50	154 (19.2)
51–60	30 (3.7)
> 60	1 (0.1)
Educational level	
Junior college degree	19 (2.4)
Bachelor’s degree	232 (28.9)
Master’s degree	281 (35.0)
Doctor’s degree	272 (33.8)
Professional title	
Junior	249 (31.0)
Intermediate	342 (42.5)
Associate senior	157 (19.5)
Senior	56 (7.0)
Department	
Surgical department	259 (32.2)
Non-surgical department	310 (38.6)
Medical technology department	149 (18.5)
Management department	86 (10.7)
Current research type	
Clinical research	306 (38.1)
Laboratory research	51 (6.3)
Clinical and laboratory research	217 (27.0)
Public health management research	41 (5.1)
Do not conduct research	189 (23.5)
Role in current research	
National PI	86 (10.7)
Provincial PI	58 (7.2)
City-level PI	60 (7.5)
Department-level PI	49 (6.1)
Project participants	304 (37.8)
No project support	247 (30.7)
Current research pressure	
Very low	31 (3.9)
Low	38 (4.7)
Moderate	206 (25.6)
High	376 (46.8)
Very high	153 (19.0)
Preferred collaboration partners at the institutional or individual level	
Institutions	380 (47.3)
Individuals	413 (51.4)
Uncertain	11 (1.4)
Preferred partners of collaboration in the research application stage	
University	585 (72.8)
Hospital	386 (48.0)
Research institute	586 (72.9)
Industry	225 (28.0)
Community	106 (13.2)
Preferred partners of collaboration in the research implementation stage	
University	430 (53.5)
Hospital	488 (60.7)
Research institute	573 (71.3)
Industry	344 (42.8)
Community	174 (21.6)
Preferred partners of collaboration in the research achievement promotion stage	
University	324 (40.3)
Hospital	453 (56.3)
Research institute	388 (48.3)
Industry	555 (69.0)
Community	354 (44.0)
Preferred partners of collaboration in the research application stage	
Laboratory scientist	632 (78.6)
Clinical scientist	579 (72.0)
Industrial staff	225 (28.0)
Community staff	159 (19.8)
Health management scientist	213 (26.5)
Preferred partners of collaboration in the research implementation stage	
Laboratory scientist	516 (64.2)
Clinical scientist	620 (77.1)
Industrial staff	357 (44.4)
Community staff	260 (32.3)
Health management scientist	258 (32.1)
Preferred partners of collaboration in the research achievement promotion stage	
Laboratory scientist	334 (41.5)
Clinical scientist	470 (58.5)
Industrial staff	542 (67.4)
Community staff	434 (54.0)
Health management scientist	431 (53.6)
Preferred research relationship	
Independent	406 (50.5)
Interdependent	398 (49.5)

**Table 2:** Basic characteristics of participating clinicians

Characteristic	N (%)				
**Communication methods used in collaboration**	**Almost no**	**Seldom**	**Sometimes**	**Often**	**Always**
Face-to-face	65 (8.1)	114 (14.2)	299 (37.2)	266 (33.1)	60 (7.5)
Telephone	59 (7.3)	85 (10.6)	239 (29.7)	345 (42.9)	76 (9.5)
WeChat	57 (7.1)	52 (6.5)	182 (22.6)	406 (50.5)	107 (13.3)
Email	83 (10.3)	115 (14.3)	244 (30.3)	289 (35.9)	73 (9.1)
Research record, research abstract, memorandum	93 (11.6)	108 (13.4)	246 (30.6)	280 (34.8)	77 (9.6)
Videoconference	113 (14.1)	128 (15.9)	259 (32.2)	248 (30.8)	56 (7.0)
Face-to-face group meeting	82 (10.2)	116 (14.4)	292 (36.3)	258 (32.1)	56 (7.0)
Mobile short message	118 (14.7)	118 (14.7)	248 (30.8)	267 (33.2)	53 (6.6)
**Preferred partners of collaboration**	**Strongly disagree**	**Disagree**	**Uncertain**	**Agree**	**Strongly agree**
University	2 (0.2)	4 (0.5)	47 (5.8)	465 (57.8)	286 (35.6)
Hospital	2 (0.2)	10 (1.2)	80 (10.0)	479 (59.6)	233 (29.0)
Research institute	1 (0.1)	1 (0.1)	36 (4.5)	479 (59.6)	287 (35.7)
Industry	2 (0.2)	32 (4.0)	202 (25.1)	390 (48.5)	178 (22.1)
Community	8 (1.0)	50 (6.2)	225 (28.0)	374 (46.5)	147 (18.3)
**Advantages of collaboration**	**Strongly disagree**	**Disagree**	**Uncertain**	**Agree**	**Strongly agree**
Additional funds or resources	7 (0.9)	9 (1.1)	102 (12.7)	453 (56.3)	233 (29.0)
Promoted knowledge transfer	7 (0.9)	5 (0.6)	46 (5.7)	482 (60.0)	264 (32.8)
Enhanced institution reputation	6 (0.7)	6 (0.7)	80 (10.0)	471 (58.6)	241 (30.0)
Increased number of publications	5 (0.6)	3 (0.4)	50 (6.2)	474 (59.0)	272 (33.8)
Improved publication quality	4 (0.5)	2 (0.2)	46 (5.7)	464 (57.7)	288 (35.8)
Enriched academic influence	4 (0.5)	3 (0.4)	43 (5.3)	475 (59.1)	279 (34.7)
Additional clinical resources	6 (0.7)	8 (1.0)	53 (6.6)	463 (57.6)	274 (34.1)
More equipment resources	5 (0.6)	8 (1.0)	61 (7.6)	456 (56.7)	274 (34.1)
New technologies	4 (0.5)	3 (0.4)	64 (8.0)	470 (58.5)	263 (32.7)
Promoted treatment capability	5 (0.6)	3 (0.4)	71 (8.8)	468 (58.2)	257 (32.0)
Accelerated research process	4 (0.5)	6 (0.7)	51 (6.3)	457 (56.8)	286 (35.6)
**Extra resources made available through collaboration**	**Strongly disagree**	**Disagree**	**Uncertain**	**Agree**	**Strongly agree**
Funds	4 (0.5)	17 (2.1)	132 (16.4)	474 (59.0)	177 (22.0)
Patients	6 (0.7)	23 (2.9)	107 (13.3)	475 (59.1)	193 (24.0)
Technologies	4 (0.5)	4 (0.5)	44 (5.5)	494 (61.4)	258 (32.1)
Equipment	4 (0.5)	5 (0.6)	56 (7.0)	492 (61.2)	247 (30.7)
Talents	5 (0.6)	8 (1.0)	83 (10.3)	487 (60.6)	221 (27.5)
Information	5 (0.6)	3 (0.4)	44 (5.5)	507 (63.1)	245 (30.5)
**Personal capabilities influenced by collaboration**	**Strongly disagree**	**Disagree**	**Uncertain**	**Agree**	**Strongly agree**
Improved skills of communication	2 (0.2)	3 (0.4)	59 (7.3)	543 (67.5)	197 (24.5)
Improved skills of receiving new knowledge and technology	2 (0.2)	2 (0.2)	55 (6.8)	522 (64.9)	223 (27.7)
Improved skills of controlling over research programs	1 (0.1)	8 (1.0)	115 (14.3)	503 (62.6)	177 (22.0)
**Disadvantages of collaboration**	**Strongly disagree**	**Disagree**	**Uncertain**	**Agree**	**Strongly agree**
More time spent on communication	9 (1.1)	74 (9.2)	171 (21.3)	469 (58.3)	81 (10.1)
Personal resource transfer	12 (1.5)	99 (12.3)	210 (26.1)	418 (52.0)	65 (8.1)
Loss of research autonomy and control	11 (1.4)	103 (12.8)	219 (27.2)	382 (47.5)	89 (11.1)
Deviation from one’s main research	14 (1.7)	103 (12.8)	262 (32.6)	358 (44.5)	67 (8.3)
Conflicts regarding key research points	10 (1.2)	69 (8.6)	242 (30.1)	403 (50.1)	80 (10.0)
**Costs of collaboration**	**Strongly disagree**	**Disagree**	**Uncertain**	**Agree**	**Strongly agree**
Costs of selecting partners and collecting information	2 (0.2)	6 (0.7)	74 (9.2)	572 (71.1)	150 (18.7)
Costs of negotiation	1 (0.1)	10 (1.2)	106 (13.2)	536 (66.7)	151 (18.8)
Costs of implementation	2 (0.2)	5 (0.6)	86 (10.7)	552 (68.7)	159 (19.8)
Costs of supervision	1 (0.1)	7 (0.9)	119 (14.8)	522 (64.9)	155 (19.3)
**Risks of collaboration**	**Strongly disagree**	**Disagree**	**Uncertain**	**Agree**	**Strongly agree**
Risks of coordinating the relationship among all partners	2 (0.2)	5 (0.6)	98 (12.2)	561 (69.8)	138 (17.2)
Risks of having unbalanced duties and responsibilities undertaken by different partners	1 (0.1)	5 (0.6)	94 (11.7)	543 (67.5)	161 (20.0)
Risks of dropping out or breaking of promises by partners	1 (0.1)	10 (1.2)	108 (13.4)	512 (63.7)	173 (21.5)
**Challenges of collaboration**	**Strongly disagree**	**Disagree**	**Uncertain**	**Agree**	**Strongly agree**
Competition from other research organizations	2 (0.2)	12 (1.5)	115 (14.3)	519 (64.6)	156 (19.4)
Ethics review process	2 (0.2)	30 (3.7)	135 (16.8)	505 (62.8)	132 (16.4)
Insufficient research funds	1 (0.1)	20 (2.5)	145 (18.0)	497 (61.8)	141 (17.5)
Recruitment of project managers	3 (0.4)	4 (0.5)	94 (11.7)	506 (62.9)	197 (24.5)
**Factors influencing collaboration**	**Strongly disagree**	**Disagree**	**Uncertain**	**Agree**	**Strongly agree**
Geographical locations of partners	5 (0.6)	20 (2.5)	105 (13.1)	517 (64.3)	157 (19.5)
Funds of partners	1 (0.1)	11 (1.4)	121 (15.0)	501 (62.3)	170 (21.1)
Technologies of partners	2 (0.2)	6 (0.7)	50 (6.2)	496 (61.7)	250 (31.1)
Information resources of partners	2 (0.2)	5 (0.6)	56 (7.0)	502 (62.4)	239 (29.7)
Academic status of partners	2 (0.2)	6 (0.7)	84 (10.4)	495 (61.6)	217 (27.0)
Mutual relationships of partners	1 (0.1)	8 (1.0)	92 (11.4)	495 (61.6)	208 (25.9)
Cooperation patterns of partners	1 (0.1)	4 (0.5)	69 (8.6)	529 (65.8)	201 (25.0)
**Factors influencing successful collaboration**	**Very unimportant**	**Unimportant**	**Moderate**	**Important**	**Very important**
Set explicit collaboration aims	2 (0.2)	1 (0.1)	50 (6.2)	493 (61.3)	258 (32.1)
Set specific collaboration periods	3 (0.4)	4 (0.5)	94 (11.7)	506 (62.9)	197 (24.5)
Choose appropriate partners	1 (0.1)	2 (0.2)	50 (6.2)	480 (59.7)	271 (33.7)
Establish clear collaboration rules	1 (0.1)	5 (0.6)	60 (7.5)	492 (61.2)	246 (30.6)
Set clear-cut benefit distribution rules	2 (0.2)	8 (1.0)	91 (11.3)	464 (57.7)	239 (29.7)
Set explicit risk-taking rules	1 (0.1)	3 (0.4)	90 (11.2)	474 (59.0)	236 (29.4)
Maintain cooperative network relationships	1 (0.1)	8 (1.0)	117 (14.6)	497 (61.8)	181 (22.5)
Establish coordination and supervision mechanisms	1 (0.1)	5 (0.6)	95 (11.8)	488 (60.7)	215 (26.7)
Set penalty rules for violations of the collaboration agreement	2 (0.2)	10 (1.2)	114 (14.2)	475 (59.1)	203 (25.2)
Set rules for dealing with disputes or emergencies	2 (0.2)	4 (0.5)	93 (11.6)	488 (60.7)	217 (27.0)

### Collaborative willingness in TMR among clinicians

#### Univariate analysis of influential factors

Of the 804 participants, 429 (53.4%) were willing and 304 (37.8%) were very willing to collaborate in TMR, while 14 (1.7%) were unwilling and 57 (7.1%) were uncertain. To explore the relationships between the various factors and clinicians’ collaborative willingness in TMR, we performed nonparametric tests. The results (Tables 3 and 4) indicated that 25 factors were statistically significant; these include clinicians’ age, education level, professional title, current research type, role in the current TMR, present research pressure, face-to-face communication in collaboration, preferred collaboration partners at the institutional or individual level, and preferences for independent or interdependent relationships. The recognized advantages of collaboration; extra resources brought by collaboration; personal capabilities that might be improved, and perceived disadvantages; costs, risks, and challenges of collaboration; and the factors related to the implementation and success of the collaboration were also factors (all *p-*values < 0.05).

**Table 3:** Univariate analysis of factors influencing willingness to collaborate in TMR among clinicians

Characteristic	Willingness of collaboration in TMR					Statistic	*P*-value
	Very unwilling	Unwilling	Uncertain	Willing	Very willing		
	9	5	57	429	304		
Sex						− 1.479	0.139
Male	3	3	30	243	183		
Female	6	2	27	186	121		
Age (year)						9.562	0.049*
20–30	3	1	26	117	73		
31–40	2	3	17	219	158		
41–50	3	1	9	77	64		
51–60	1	0	5	15	9		
> 60	0	0	0	1	0		
Educational level						22.161	< 0.0001*
Junior college degree	0	1	4	7	7		
Bachelor’s degree	5	1	33	123	70		
Master’s degree	3	3	10	159	106		
Doctor’s degree	1	0	10	140	121		
Professional title						10.213	0.017*
Junior	3	2	32	128	84		
Intermediate	3	3	17	192	127		
Associate senior	1	0	4	88	64		
Senior	2	0	4	21	29		
Department						5.685	0.128
Surgical department	1	2	14	134	108		
Non-surgical department	4	2	21	175	108		
Medical technology department	2	0	8	82	57		
Management department	2	1	14	38	31		
Current research type						27.285	< 0.0001*
Clinical research	1	2	15	179	109		
Laboratory research	0	0	0	27	24		
Clinical and laboratory research	3	0	7	109	98		
Public health management research	1	0	2	21	17		
Do not conduct research	4	3	33	93	56		
Role in current research						34.147	< 0.0001*
National PI	0	0	2	38	46		
Provincial PI	1	0	0	28	29		
City-level PI	1	0	2	25	32		
Department-level PI	1	1	3	22	22		
Project participants	1	1	13	196	93		
No project support	5	3	37	120	82		
Current research pressure						44.411	< 0.0001*
Very low	1	3	9	9	9		
Low	0	1	2	25	10		
Moderate	5	0	21	123	57		
High	2	1	18	214	141		
Very high	1	0	7	58	87		
Communication methods used in collaboration							
Face-to-face	9	5	57	429	304	19.615	0.001*
Telephone	9	5	57	429	304	6.415	0.170
WeChat	9	5	57	429	304	13.868	0.008
Email	9	5	57	429	304	5.748	0.219
Research record, research abstract, memorandum	9	5	57	429	304	2.710	0.608
Videoconference	9	5	57	429	304	6.342	0.175
Face-to-face group meeting	9	5	57	429	304	4.749	0.314
Mobile short message	9	5	57	429	304	5.133	0.274
Preferred collaboration partners at the institutional or individual level						39.784	< 0.0001*
Institutions	3	0	22	190	165		
Individuals	4	3	29	238	139		
Uncertain	2	2	6	1	0		
Preferred partners of collaboration							
University	9	5	57	429	304	64.543	< 0.0001*
Hospital	9	5	57	429	304	43.938	< 0.0001*
Research institute	9	5	57	429	304	68.770	< 0.0001*
Industry	9	5	57	429	304	33.436	< 0.0001*
Community	9	5	57	429	304	19.394	0.001*
Preferred partners of collaboration in the research application stage							
University	7	2	31	297	248	− 4.853	< 0.0001*
Hospital	5	2	25	184	170	3.176	0.002*
Research institute	7	2	38	301	238	− 2.764	0.006*
Industry	4	1	15	103	102	2.326	0.020*
Community	2	1	9	43	51	1.672	0.095*
Preferred partners of collaboration in the research implementation stage							
University	5	3	22	201	199	− 5.217	< 0.0001*
Hospital	6	2	28	247	205	− 3.225	0.001*
Research institute	6	2	38	294	233	− 2.702	0.007*
Industry	5	0	18	169	152	− 16.779	< 0.0001*
Community	2	1	9	72	90	4.011	< 0.0001*
Preferred partners of collaboration in the research achievement promotion stage							
University	3	2	17	157	145	3.431	0.001*
Hospital	8	2	26	223	194	− 3.166	0.002*
Research institute	4	1	29	193	161	1.828	0.068
Industry	6	2	29	292	226	− 3.277	0.001*
Community	4	1	19	169	161	4.035	< 0.0001*
Preferred partners of collaboration in the research application stage							
Laboratory scientist	5	2	37	331	257	− 3.998	< 0.0001*
Clinical scientist	8	1	38	301	231	− 2.070	0.039*
Industrial staff	4	0	18	102	101	2.015	0.044*
Community staff	4	2	17	74	62	− 0.686	0.493
Health management scientist	3	1	13	87	109	4.194	< 0.0001*
Preferred partners of collaboration in the research implementation stage							
Laboratory scientist	4	1	32	268	211	− 2.859	0.004*
Clinical scientist	1	3	17	101	62	− 1.601	0.109
Industrial staff	5	1	27	174	150	1.765	0.078
Community staff	4	1	19	127	109	0.925	0.355
Health management scientist	4	1	12	125	116	2.973	0.003*
Preferred partners of collaboration in the achievement promotion stage							
Laboratory scientist	3	1	25	166	139	1.674	0.094
Clinical scientist	7	1	33	248	181	− 0.457	0.648
Industrial staff	5	1	33	275	228	− 3.899	< 0.0001*
Community staff	5	2	29	225	173	− 1.314	0.189
Health management scientist	4	1	23	211	192	− 4.501	< 0.0001*

*Indicates statistically significant results (p < 0.05)

**Table 4:** Univariate analysis of factors influencing willingness to collaborate in TMR among clinicians

Characteristic	Willingness of collaboration in TMR					Statistic	*P*-value
	Very unwilling	Unwilling	Uncertain	Willing	Very willing		
	9	5	57	429	304		
Preferred research relationship						3.326	0.001*
Independent	6	5	29	236	130		
Interdependent	3	0	28	193	174		
Advantages of collaboration							
Additional funds or resources	9	5	57	429	304	52.917	< 0.0001*
Promoted knowledge transfer	9	5	57	429	304	77.588	< 0.0001*
Enhanced institution reputation	9	5	57	429	304	53.686	< 0.0001*
Increased number of publications	9	5	57	429	304	77.190	< 0.0001*
Improved publication quality	9	5	57	429	304	72.425	< 0.0001*
Enriched academic influence	9	5	57	429	304	84.365	< 0.0001*
Additional clinical resources	9	5	57	429	304	62.820	< 0.0001*
More equipment resources	9	5	57	429	304	61.191	< 0.0001*
New technologies	9	5	57	429	304	78.946	< 0.0001*
Promoted treatment capability	9	5	57	429	304	70.646	< 0.0001*
Accelerated research process	9	5	57	429	304	92.024	< 0.0001*
Extra resources made available through collaboration							
Funds	9	5	57	429	304	25.898	< 0.0001*
Patients	9	5	57	429	304	40.559	< 0.0001*
Technologies	9	5	57	429	304	42.761	< 0.0001*
Equipment	9	5	57	429	304	40.517	< 0.0001*
Talents	9	5	57	429	304	37.620	< 0.0001*
Information	9	5	57	429	304	49.440	< 0.0001*
Personal capabilities influenced by collaboration							
Improved skills of communication	9	5	57	429	304	54.406	< 0.0001*
Improved skills of receiving new knowledge and technology	9	5	57	429	304	53.967	< 0.0001*
Improved skills of controlling over research programs	9	5	57	429	304	41.469	< 0.0001*
Disadvantages of collaboration							
More time spent on communication	9	5	57	429	304	18.015	0.001*
Personal resource transfer	9	5	57	429	304	24.200	< 0.0001*
Loss of research autonomy and control	9	5	57	429	304	33.797	< 0.0001*
Deviation from one’s main research	9	5	57	429	304	31.963	< 0.0001*
Conflicts regarding key research points	9	5	57	429	304	25.058	< 0.0001*
Costs of collaboration							
Costs of selecting partners and collecting information	9	5	57	429	304	18.997	0.001*
Costs of negotiation	9	5	57	429	304	18.080	0.001*
Costs of implementation	9	5	57	429	304	30.360	< 0.0001*
Costs of supervision	9	5	57	429	304	46.453	< 0.0001*
Risks of collaboration							
Risks of coordinating the relationship among all partners	9	5	57	429	304	16.594	0.002*
Risks of having unbalanced duties and responsibilities undertaken by different partners	9	5	57	429	304	12.497	0.014*
Risks of dropping out or breaking of promises by partners	9	5	57	429	304	26.837	< 0.0001*
Challenges of collaboration							
Competition from other research organizations	9	5	57	429	304	22.726	< 0.0001*
Ethics review process	9	5	57	429	304	22.512	0.000*
Insufficient research funds	9	5	57	429	304	24.195	< 0.0001*
Recruitment of project managers	9	5	57	429	304	28.471	< 0.0001*
Factors influencing collaboration							
Geographical locations of partners	9	5	57	429	304	15.695	0.004*
Funds of partners	9	5	57	429	304	23.437	0.000*
Technologies of partners	9	5	57	429	304	66.833	< 0.0001*
Information resources of partners	9	5	57	429	304	58.321	< 0.0001*
Academic status of partners	9	5	57	429	304	38.837	< 0.0001*
Mutual relationships of partners	9	5	57	429	304	37.916	< 0.0001*
Cooperation patterns of partners	9	5	57	429	304	37.277	< 0.0001*
Factors influencing successful collaboration							
Set explicit collaboration aims	9	5	57	429	304	65.751	< 0.0001*
Set specific collaboration periods	9	5	57	429	304	41.773	< 0.0001*
Choose appropriate partners	9	5	57	429	304	50.887	< 0.0001*
Establish clear collaboration rules	9	5	57	429	304	49.138	< 0.0001*
Set clear-cut benefit distribution rules	9	5	57	429	304	36.000	< 0.0001*
Set explicit risk-taking rules	9	5	57	429	304	45.529	< 0.0001*
Maintain cooperative network relationships	9	5	57	429	304	38.086	< 0.0001*
Establish coordination and supervision mechanisms	9	5	57	429	304	43.148	< 0.0001*
Set penalty rules for violations of the collaboration agreement	9	5	57	429	304	65.377	< 0.0001*
Set rules for dealing with disputes or emergencies	9	5	57	429	304	32.935	< 0.0001*

*Indicates statistically significant results (p < 0.05)

#### Logistic regression analysis of willingness to collaborate in TMR

Based on the results of the univariate analyses, only the factors that had a statistically significant influence on collaborative willingness in TMR among clinicians were included in the logistic regression analysis. The logistic regression analysis results (Table 5) suggested that clinicians’ current research type, role in the current TMR, present research pressure, preferred collaboration partners at the institutional or individual level, and preferences for independent or interdependent relationships were statistically significant factors. Greater willingness to collaborate in TMR was associated with clinicians who were not conducting research (compared with those engaged in clinical research, odds ratio [*OR*] = 0.424), those who were acting as project participants (compared with national PI, *OR* = 0.396), and those who were more willing to collaborate with individuals or without explicit preferences at the institutional or individual level (compared with those who were more willing to collaborate with institutions; *OR* = 0.554 and 0.011, respectively). However, less willingness to collaborate in TMR was associated with clinicians with heavier research burdens (compared with those with low burden, *OR* = 2.591), those preferring to collaborate with hospitals in the research implementation stage (compared with those without such preference, *OR* = 1.422), and those tending to opt for an interdependent research relationship (compared with those tending to be independent, *OR* = 1.495).

**Table 5:** Logistic regression analysis of the collaborative willingness in TMR

Characteristic	Estimate	Wald Chi-Square	P-value	OR	95% Wald confidence limits	
					Lower	**Upper**
Current research type						
Clinical research	Ref					
Laboratory research	0.433	1.603	0.205	1.541	0.789	3.011
Clinical and laboratory research	0.300	2.030	0.154	1.350	0.893	2.041
Public health management research	0.331	0.709	0.400	1.393	0.644	3.012
Do not conduct research	− 0.858	8.909	0.003*	0.424	0.241	0.745
Role in current research						
National PI	Ref					
Provincial PI	− 0.277	0.508	0.476	0.758	0.354	1.624
City-level PI	− 0.436	1.285	0.257	0.647	0.305	1.374
Department-level PI	− 0.583	2.041	0.153	0.558	0.251	1.242
Project participants	− 0.926	10.108	0.002*	0.396	0.224	0.701
No project support	− 0.496	1.799	0.180	0.609	0.295	1.257
Current research pressure						
Very low	Ref					
Low	0.151	0.079	0.779	1.163	0.407	3.323
Moderate	0.002	0.000	0.997	1.002	0.421	2.383
High	0.253	0.333	0.564	1.288	0.545	3.043
Very high	0.952	4.247	0.039*	2.591	1.048	6.406
Preferred collaboration partners at the institutional or individual level						
Institutions	Ref					
Individuals	− 0.591	13.579	0.00*	0.554	0.405	0.758
Uncertain	− 4.545	42.277	< 0.0001*	0.011	0.003	0.042
Willing to collaborate with industry						
Strongly agree	Ref					
Agree	3.142	1.892	0.169	23.156	0.263	> 999.999
Uncertain	2.965	1.722	0.190	19.389	0.231	> 999.999
Agree	3.534	2.451	0.118	34.250	0.411	> 999.999
Strongly agree	3.143	1.969	0.161	23.168	0.287	> 999.999
Preference for collaborating with hospitals in the research implementation stage						
No	Ref					
Yes	0.352	4.241	0.040*	1.422	1.017	1.988
Preference for collaborating with community in the research achievement promotion stage						
No	Ref					
Yes	0.332	3.691	0.055	1.393	0.993	1.955
Preference for collaborating with health management scientists in the research application stage						
No	Ref					
Yes	0.367	3.575	0.059	1.444	0.987	2.112
Preferred research relationship						
Independent	Ref					
Interdependent	0.402	6.175	0.013*	1.495	1.089	2.052
Receive additional funds or resources by collaboration						
Strongly agree	Ref					
Agree	− 2.433	0.707	0.400	0.088	< 0.001	25.436
Uncertain	− 2.529	0.806	0.369	0.080	< 0.001	19.934
Agree	− 1.716	0.374	0.541	0.180	< 0.001	43.947
Strongly agree	− 1.992	0.502	0.479	0.136	< 0.001	33.769
Increase number of publications by collaboration						
Strongly agree	Ref					
Agree	2.641	0.537	0.464	14.024	0.012	> 999.999
Uncertain	− 1.847	0.366	0.545	0.158	< 0.001	62.343
Agree	− 1.297	0.183	0.669	0.273	< 0.001	103.818
Strongly agree	− 0.964	0.102	0.750	0.381	0.001	143.427
Accelerate research process by collaboration						
Strongly agree	Ref					
Agree	1.518	0.202	0.653	4.562	0.006	> 999.999
Uncertain	3.789	1.248	0.264	44.205	0.057	> 999.999
Agree	3.325	0.971	0.324	27.790	0.037	> 999.999
Strongly agree	4.427	1.707	0.191	83.673	0.109	> 999.999
Extra funds made available through collaboration						
Strongly agree	Ref					
Agree	1.447	0.249	0.618	4.252	0.014	> 999.999
Uncertain	0.314	0.012	0.912	1.368	0.005	360.494
Agree	− 0.048	0.000	0.987	0.953	0.004	245.458
Strongly agree	− 0.515	0.033	0.856	0.598	0.002	156.467
Improve skills of communication by collaboration						
Strongly agree	Ref					
Agree	− 0.433	0.015	0.904	0.649	< 0.001	733.327
Uncertain	− 0.015	0.000	0.997	0.985	0.001	718.965
Agree	− 0.503	0.022	0.881	0.605	< 0.001	439.175
Strongly agree	0.330	0.010	0.922	1.390	0.002	999.381
Lose research autonomy and control by collaboration						
Strongly agree	Ref					
Agree	0.387	0.207	0.549	1.472	0.279	7.775
Uncertain	− 0.192	0.053	0.818	0.825	0.161	4.233
Agree	− 0.424	0.261	0.610	0.654	0.128	3.336
Strongly agree	− 0.120	0.020	0.887	0.887	0.169	4.644

*Indicates statistically significant results (p < 0.05)

## Discussion

The results of our survey indicated that clinicians’ current research situation—including the research type, role, and pressure—preferred partners, and preferences for the research relationship were key factors that influenced their willingness to collaborate in TMR. Moreover, clinician’s cognition on translational medicine has an impact on their willingness to collaborate in TMR. Clinicians with more positive attitudes and more knowledge will be more likely to take measures in collaborating in TMR.

Those who were not engaged in any research at the time and who were participants in current research programs were more willing to collaborate in TMR, which could be explained by their research pressure or lack thereof. Collaborations in TMR would impose an additional research burden on clinicians, as they would be required to spend more time in communication and resource reallocation [32]. Therefore, clinicians with heavy research loads would be less likely to collaborate in TMR, while those with more time and fewer research responsibilities would be more willing to collaborate. In addition, considering the advantages of TMR collaboration in terms of promoting knowledge transfer and increasing research achievements, clinicians not currently involved in any research or whose involvement was only as a participant would seek more knowledge exchange and increased research achievements through collaboration, which represent incentives for collaboration in TMR [3]. Considering the disadvantages of collaboration, it was shown to weaken control over the research program, which was unacceptable for those undertaking national research projects because such a disadvantage outweighed the merits mentioned above. It is worth noting that some demographic characteristics—such as age, education level, and professional title—would have an indirect effect on clinicians’ willingness to collaborate. For example, younger clinicians and those with junior professional titles would be more willing to collaborate due to having more time and fewer research responsibilities as well as for possible knowledge transfer and gaining research achievements. Clinicians with a higher education level would be more willing to collaborate because they may have greater demands for and possibilities of knowledge exchange and research achievements.

Clinicians who preferred individuals as partners, rather than institutions, were more willing to collaborate in TMR. Institutional arrangements are perceived to inhibit collaboration [13], with the compartmentalization of institutions being a main obstruction to collaboration [7]. Therefore, considering the uncertainty of the potential cooperative institutions, clinicians may be inclined to think that collaborating with individuals would avoid risks such as inappropriate institutional arrangements and structural isolation among different institutions. Moreover, face-to-face communication among clinicians who choose to collaborate with individuals is more likely, which would further promote their willingness to collaborate with individuals. In terms of the benefits of collaborating, working with individuals would also help individual scientists expand the scope and sample size of their research and improve efficiency [9], which is another motivation for collaborating with individuals. Further, considering the preferred institution in the research implementation phase, clinicians who preferred to collaborate with hospitals tended to be more unwilling to collaborate in TMR. A previous study indicated that enhancing the impact of clinical therapy and policy is the core objective of TMR [8]. However, realizing this goal is not easy and it can be time consuming. Such delays may lead to changes, beyond expectations, after collaboration, which consequently impede collaboration in TMR [3]. Thus, although the original aim of collaborating with hospitals was to increase influence by accelerating the clinical application process of research achievements, it would cost more time to realize such translation, which could lead to higher potential risks. The gap between reality and the ideal circumstances would undermine these clinicians’ willingness to collaborate.

Clinicians who are inclined to be independent in research relationships were more willing to collaborate in TMR. Our results confirmed those of other studies and suggested that the advantages of collaboration would not promote collaboration in TMR [3]. Instead, clinicians who are inclined to be in independent research relationships were less likely to depend on benefits from collaboration, and this tendency facilitated their collaborative willingness. In addition, clinicians who prefer an independent research relationship were more receptive to competing with partners for funds, talent, reputation, and extra support. Such effective and positive competition among partners would promote creativity and excellence [32], which was another form of encouragement for these clinicians to collaborate in TMR.

The results of our study showed that the factors that influenced collaboration in TMR primarily concerned current research characteristics, collaborative partners, and relationships with partners. The advantages and disadvantages of collaboration were not the main concerns for clinicians when deciding upon collaboration; this indirectly demonstrated that the decision to collaborate was dependent on the feasibility of collaboration (including the availability of time and suitable partners), and that most of the advantages and disadvantages were acceptable, meaning that no additional consideration was required. However, to promote collaboration in TMR, such factors mentioned above should be taken into consideration, which will be helpful to choose more suitable partners and take favorable measures at each stage of collaboration.

The present study has certain limitations. First, most questions in our internet-based survey were self-reported, a method that is prone to potential over- or under-estimation. Second, the study did not distinguish between multi-team systems collaboration and individual collaboration. The factors that influence multi-team systems collaboration and individual collaboration may differ; we will further explore this issue in a follow-up study. Third, we did not consider the role of innovation intermediaries in collaborative TMR. We will explore how innovation intermediaries affect collaborations among clinicians in translational medicine in subsequent studies. Fourth, our survey ignored the leadership role of different agents in TMR. In further surveys, if the preferred partner was an individual (laboratory scientist, clinical scientist, industrial staff, community staff, or health management scientist), the leadership role of clinicians or these individuals should be investigated. If the preferred partner was an institution (university, hospital, research institute, industry, or community) the leadership role of clinicians or these institutions should also be considered.

## Conclusions

Most Chinese clinicians who were enrolled in this study are willing to collaborate in TMR. Their willingness to collaborate was mainly based on the current TMR characteristics, potential partners, and inclinations regarding research relationships. To collaborate appropriately and foster the greatest advantages of collaboration in TMR, clinicians should seriously consider the advantages and disadvantages of collaboration. First, clinicians should evaluate their time, role, and research pressure based on their current TMR before deciding to collaborate. Personal preferences for research relationships should also be considered. Second, clinicians should fully consider the goals of their TMR during the entire process and in the different research phases, which would help them choose appropriate partners and address the various costs, risks, and challenges involved in collaborations. Third, several measures could be taken at the national level to accelerate collaboration in TMR, including supporting more resources (e.g., funds), providing training and education programs on the implementation and management of TMR, and giving specific regulations on the responsibilities and rights among partners in TMR.

## Data Availability

The datasets used and/or analysed during the current study are available from the corresponding author on reasonable request.

## Abbreviations

TMR: Translational medical research
PI: Principal Investigator
